# Positive fluid balance is an independent risk factor for acute kidney injury in critically ill patients: results of a prospective, cross-sectional study

**DOI:** 10.1186/cc14270

**Published:** 2015-03-16

**Authors:** N Salahuddin, M Sammani, A Hamdan, M Joseph, Y AlNemary, R Alquaiz, K Maghrabi

**Affiliations:** 1 King Faisal Specialist Hospital & Research Centre, Riyadh, Saudi Arabia

## Introduction

In critical illness, fluid overload may predispose to acute renal dysfunction by a number of mechanisms. Once acute kidney injury (AKI) develops, positive fluid balance has been described as a risk factor for overall mortality and delayed renal recovery. We hypothesized that fluid overload may be an independent risk factor for AKI in the critically ill.

## Methods

In a cross-sectional design, we collected data on consecutive, critically ill, adult patients admitted over a 5-month period to the medical and surgical ICUs of a single center. AKI was defined according to the RIFLE Classification. Logistic regression analysis was performed to determine the predictive ability of variables for AKI. The institutional Research Ethics Committee approved the study.

## Results

Three hundred and thirty-nine patients were included; mean age was 51 ± 20.4 years, 167 (49%) patients were male. Mean APACHE II score was 22 ± 12.8 and SAPS II score was 35.4 ± 18.9. Severe sepsis/ septic shock was the admitting diagnosis in 129 (38%) patients, 108 (32%) patients were postoperative. AKI developed in 148 (44%) patients; Risk 29 (9%); Injury 26 (8%); Failure 89 (26%) by the RIFLE criteria. On univariate regression analysis; positive fluid balance >2 l on the first ICU admission day, OR 2 (95% CI = 1.3, 3.3, *P *= 0.002); age, OR 2.7 (95% CI = 1.7, 4.2, *P *= 0.000); CHF, OR 3.1 (95% CI = 1.2, 7.9, *P *= 0.013); APACHE II score, OR 1.02 (95% CI = 1.0, 1.04, *P *= 0.006); SAPS II score, OR 1.04 (95% CI = 1.02, 1.05, *P *= 0.000); mean MAP on admission OR 0.98 (95% CI = 0.96, 0.99, *P *= 0.033); need for vasopressors on admission, OR 2.7 (95% CI = 1.7, 4.2, *P *< 0.001) and for >24 hours, OR 2.7 (95% CI = 1.7, 2.5, *P *< 0.001); vancomycin use, OR 1.5 (95% CI = 1.02, 2.53, *P *= 0.04) significantly predicted the development of AKI. On multivariate regression, CHF, OR 3.8 (95% CI = 1.4, 10, *P *= 0.007); age, OR 1.02 (95% CI = 1.01, 1.03, *P *= 0.001); vasopressors for >24 hours, OR 2.6 (95% CI = 1.6, 4.2, *P *< 0.001) and a >2 l positive fluid balance on the first ICU day, OR 1.6 (95% CI = 1.02, 2.7, *P *= 0.04) remained significant predictors.

## Conclusion

Fluid overload, defined as a >2 l positive fluid balance on the first day of ICU admission, is an independent risk factor for the development of AKI in the general ICU population.

